# Overexpression of GAB2 in ovarian cancer cells promotes tumor growth and angiogenesis by upregulating chemokine expression

**DOI:** 10.1038/onc.2015.472

**Published:** 2015-12-14

**Authors:** C Duckworth, L Zhang, S L Carroll, S P Ethier, H W Cheung

**Affiliations:** 1 Department of Pathology and Laboratory Medicine, Medical University of South Carolina, Charleston, SC, USA; 2 Hollings Cancer Center, Medical University of South Carolina, Charleston, SC, USA; 3 Center for Genomic Medicine, Medical University of South Carolina, Charleston, SC, USA

## Abstract

We previously found that the scaffold adapter GRB2-associated binding protein 2 (GAB2) is amplified and overexpressed in a subset of primary high-grade serous ovarian cancers and cell lines. Ovarian cancer cells overexpressing GAB2 are dependent on GAB2 for activation of the phosphatidylinositol 3-kinase (PI3K) pathway and are sensitive to PI3K inhibition. In this study, we show an important role of GAB2 overexpression in promoting tumor angiogenesis by upregulating expression of multiple chemokines. Specifically, we found that suppression of GAB2 by inducible small hairpin RNA in ovarian cancer cells inhibited tumor cell proliferation, angiogenesis and peritoneal tumor growth in immunodeficient mice. Overexpression of GAB2 upregulated the secretion of several chemokines from ovarian cancer cells, including CXCL1, CXCL2 and CXCL8. The secreted chemokines not only signal through endothelial CXCR2 receptor in a paracrine manner to promote endothelial tube formation, but also act as autocrine growth factors for GAB2-induced transformation of fallopian tube secretory epithelial cells and clonogenic growth of ovarian cancer cells overexpressing GAB2. Pharmacological inhibition of inhibitor of nuclear factor kappa-B kinase subunit β (IKKβ), but not PI3K, mechanistic target of rapamycin (mTOR) or mitogen-activated protein kinase (MEK), could effectively suppress GAB2-induced chemokine expression. Inhibition of IKKβ augmented the efficacy of PI3K/mTOR inhibition in suppressing clonogenic growth of ovarian cancer cells with GAB2 overexpression. Taken together, these findings suggest that overexpression of GAB2 in ovarian cancer cells promotes tumor growth and angiogenesis by upregulating expression of CXCL1, CXCL2 and CXCL8 that is IKKβ-dependent. Co-targeting IKKβ and PI3K pathways downstream of GAB2 might be a promising therapeutic strategy for ovarian cancer that overexpresses GAB2.

## Introduction

Ovarian cancer is the most lethal gynecological cancer, causing >14 000 deaths each year in the United States alone. Ovarian cancers are a heterogeneous group of neoplasms. Aside from being classified into different histologic subtypes, increasing evidence suggests that they can be broadly classified into two subtypes based on clinicopathological and genetic features.^1^ Type I tumors (low-grade serous, mucinous, endometriod, clear cell) are generally low-grade, localized to the ovary at diagnosis and have an indolent disease course and a better prognosis.^1^ They lack mutations of *TP53* but have frequent mutations in *KRAS*, *PIK3CA* or *BRAF* depending on the histologic subtype.^1^ By contrast, type II tumors (high-grade serous, undifferentiated cancers, carcinosarcomas) are high-grade, highly aggressive, mostly have widespread disease at presentation and thus have a poor prognosis.^1^ They have a high frequency of mutations in *TP53* and *BRCA1/2* but very rare mutations of genes that are detected in type I tumors.^1^ High-grade serous ovarian cancers (HGSOCs) represent typical type II tumors and are the most aggressive subtype that accounts for ~70% of all ovarian cancer deaths.^2^ Recent large-scale efforts by the Cancer Genome Atlas show that ovarian cancer genomes are characterized by widespread recurrent copy number alterations.^3^ Identifying and characterizing the driver genes targeted by these alterations will provide insights into the development of novel therapeutic strategies for this aggressive disease.

We previously assessed 455 genes that are significantly amplified in HGSOCs for the ability to promote tumor growth using a multiplexed open-reading frame (ORF)-based expression assay, and identified the GRB2-associated binding protein 2 (GAB2) as a putative oncogene.^4^ The chromosome 11q14.1 region involving *GAB2* is highly amplified in 14% of 562 primary HGSOCs characterized in the Cancer Genome Atlas project.^4^ Moreover, immunohistochemical analysis showed that GAB2 protein was overexpressed in 43 of 132 (33%) primary HGSOCs.^4^ These findings suggest that overexpression of GAB2 driven by genomic amplification or other mechanisms may have an important role in development and progression of HGSOCs.

GAB2 is a scaffold protein involved in signal transduction downstream of many receptor tyrosine kinases, cytokine receptors and antigen receptors.^5^ Upon receptor stimulation, GAB2 is tyrosyl-phosphorylated and capable of interacting with Src homology 2 domain-containing molecules such as the p85 regulatory subunit of phosphatidylinositol 3-kinase (PI3K), tyrosine phosphatase SHP2, phospholipase C gamma and CRK/CRKL, thereby regulating many biological processes including cell proliferation, survival, migration and differentiation.^5^ Overexpression of GAB2 has been shown to promote primary and metastatic tumor growth in breast cancer and melanoma.^6^ For example, transgenic mice overexpressing Gab2 display accelerated NeuT-induced mammary tumorigenesis through activation of Shp2-dependent mitogen-activated protein kinases signaling,^7^ whereas loss of Gab2 severely suppressed lung metastatic potential of NeuT-induced mammary tumors.^8^ Overexpression of GAB2 in NRAS-driven melanoma enhances tumor growth and angiogenesis by increasing mitogen-activated protein kinase kinase (MEK)-dependent vascular endothelial growth factor and hypoxia inducible factor 1, alpha subunit (HIFα) expression.^9^ Overexpression of GAB2 in ovarian cancer cells promotes cell migration and invasion by inducing PI3K-dependent zinc finger E-box binding homeobox 1 (ZEB1) expression.^10^ However, the mechanisms by which GAB2 overexpression contributes to tumorigenesis in ovarian cancer remain poorly defined.

The PI3K pathway is frequently activated in HGSOCs^11^ (often being described as PI3Kness) and associated with resistance to chemotherapy.^12^ As mutation of *PIK3CA* is rare (<1%), the observed PI3K pathway activation might be driven by other alterations such as loss of phosphatase and tensin homolog.^13^ We and others have shown that ovarian cancer cell lines overexpressing GAB2 are dependent on GAB2 for PI3K pathway activation and are sensitive to PI3K inhibition but not MEK inhibition,^4, 14^ suggesting GAB2 overexpression as a mechanism contributing to the PI3Kness in HGSOCs.

Although targeting PI3K pathway holds great promise for treating ovarian cancer, substantial tumor regression has not been observed in early clinical trials with inhibitors against the PI3K/AKT/mechanistic target of rapamycin (mTOR) pathway.^15, 16^ It has been suggested that inhibition of additional signaling pathways may be required to increase its efficacy.^17^ The nuclear factor-κB (NF-κB) pathway is frequently activated in ovarian cancer, and increased expression of its transcriptional targets have been associated with aggressiveness of ovarian cancer.^18, 19^ Chemokines CXCL1 and CXCL8 are well established NF-κB target genes that are frequently upregulated in serum, ascites, and tumors and are associated with poor survival in patients with ovarian cancers.^20, 21^ These chemokines could function as autocrine and paracrine growth factors for ovarian cancer.^20, 21^

In this study, we investigated the role of GAB2 overexpression in tumorigenesis of ovarian cancer. We obtained evidence that overexpression of GAB2 in ovarian cancer cells increased expression of multiple chemokines. We evaluated the efficacy of several small-molecule inhibitors against inhibitor of nuclear factor kappa-B kinase subunit β (IKKβ) on suppressing GAB2-induced chemokine expression and showed that combinatorial inhibition of IKKβ and PI3K/mTOR was more effective in suppressing proliferation and survival of GAB2-dependent ovarian cancer cells than individual inhibition.

## Results

### Suppression of GAB2 in ovarian cancer cells inhibits tumor growth and angiogenesis

**Figure 1:**
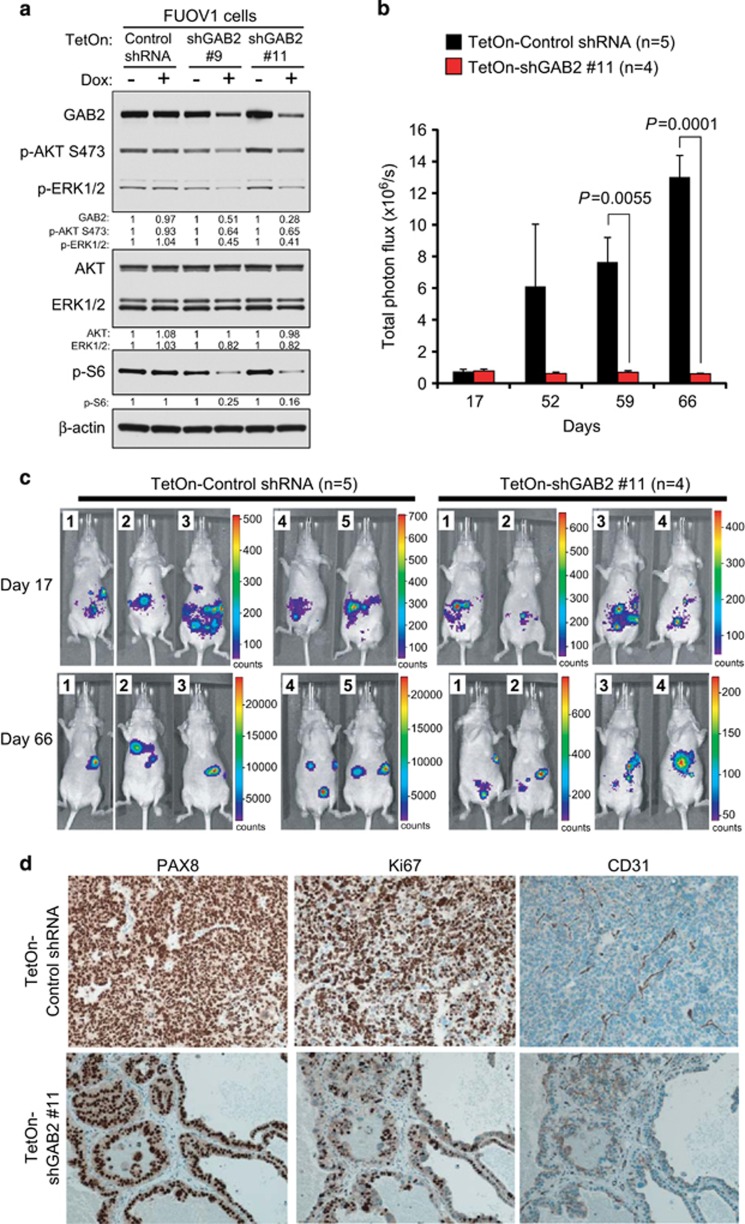
Suppression of GAB2 inhibits ovarian tumor growth. (**a**) Generation of FUOV1 ovarian cancer cell lines expressing doxycycline (Dox)-inducible control shRNA or GAB2-targeting shRNAs. FUOV1 cells expressing luciferase (FUOV1+Luc) were stably transduced with lentiviruses expressing a control shRNA or two GAB2-targeting shRNAs that were under the control of Dox-inducible promoter. Cells were cultured in the absence or presence of 1 μg/ml Dox for 96 h with the last 24 h cultured in serum-free media. Immunoblotting for GAB2, phosphorylated (p-) AKT S473, p-ERK1/2 and p-S6 were performed. The values below the figures represent relative intensity of the bands normalized to β-actin and compared with cells without doxycycline treatment. (**b**) Effect of GAB2 suppression in FUOV1 cells on the xenograft growth. FUOV1+Luc cell lines expressing Dox-inducible control shRNA or shGAB2 #11 were implanted intraperitoneally into female athymic nude mice. After 17 days, tumor burden was monitored by bioluminescent imaging. Mice were then fed on Dox-containing diet for another 49 days. Imaging was performed on 52, 59 and 66 days post implantation. *n*=4–5. Data are averages±s.e.m. (**c**) Images showing bioluminescent signals obtained on days 17 and 66 post implantation of FUOV1+Luc cells expressing Dox-inducible control shRNA (*n*=5) or shGAB2 #11 (*n*=4), as described in **b**. Note that the range of luminescence signals and color representation was different in each panel. (**d**) Representative images showing immunohistochemistry for PAX8, Ki67 and CD31 on tumors expressing Dox-inducible control shRNA or shGAB2 #11 obtained 66 days post implantation as described in **b**.

To investigate the role of GAB2 overexpression in tumorigenesis, we examined the effect of suppressing GAB2 by inducible RNA interference on ovarian tumor growth. FUOV1 ovarian cancer cells were used in this experiment. FUOV1 cells did not harbor amplification of GAB2 but displayed overexpression of GAB2 at both mRNA and protein levels (Supplementary Figures 1a and b). Significant suppression of GAB2 protein could be achieved in FUOV1 cells using an inducible small hairpin RNA (shRNA) expression system (as described below). To enable noninvasive monitoring of intraperitoneal tumor growth by luminescent imaging, FUOV1 cells were transduced to stably express luciferase (FUOV1+Luc). We then introduced these cells with a control shRNA or two previously published shRNAs targeting GAB2 (shGAB2 #9 or #11) in vectors in which the expression of shRNA was under the control of a doxycycline-inducible promoter. Doxycycline treatment of cells containing the inducible shGAB2 #9 and #11 decreased GAB2 protein expression compared with cells grown in the absence of doxycycline or in cells expressing the control shRNA (Figure 1a). Consistent with our previously published results from direct shRNA transduction experiments,^4^ inducible suppression of GAB2 also reduced phosphorylated (p-) AKT S473, p-S6 and p-ERK1/2 levels (Figure 1a) and reduced cell proliferation 6 days post treatment with doxycycline (Supplementary Figure 2).

We implanted FUOV1+Luc cells expressing inducible control shRNA or shGAB2 #11 by intraperitoneal injection into female athymic nude mice. After 17 days, mice with comparable tumor burden, as indicated by bioluminescence signals, were fed with doxycycline-containing diet for another 7 weeks (Figures 1b and c). We found that inducible suppression of GAB2 durably impaired tumor growth as the tumor burden signals remained low throughout the induction period (Figures 1b and c). By contrast, the tumor burden highly increased in control mice (Figures 1b and c). In concordance with the observed luminescence signals, palpable tumors could be resected in control mice, whereas only microscopic tumor cells that stained positive for PAX8 were detectable by immunohistochemistry in all of the four mice inoculated with cells expressing inducible shGAB2 (Figure 1d). Furthermore, we found that suppression of GAB2 inhibited tumor cell proliferation and blood vessel formation as indicated by reduced Ki67 and CD31 staining, respectively, compared with tumors expressing the control shRNA (Figure 1d). These results suggest that overexpression of GAB2 in ovarian cancer cells promotes tumor cell proliferation, angiogenesis and peritoneal tumor growth.

### Overexpression of GAB2 in ovarian cancer cells upregulates expression of CXCL1, CXCL2 and CXCL8

**Figure 2:**
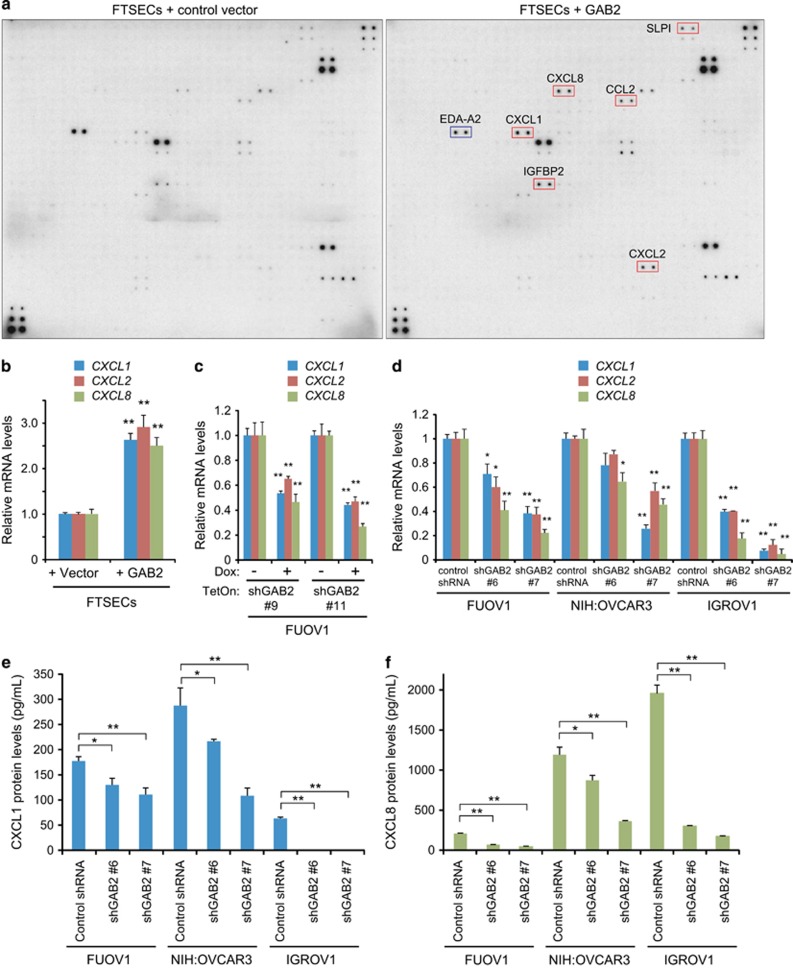
Induction of IL8, CXCL1 and CXCL2 by GAB2 overexpression. (**a**) Antibody array analyses of cell culture supernatants of FTSECs expressing a control vector or GAB2. Cells were cultured in serum-deprived media (0.1% FBS) for 48 h. Cell culture supernatants were collected and labeled with biotin before incubation with array membranes for detection. Upregulated or downregulated proteins were highlighted in red or blue, respectively. Representative images from two independent experiments were shown. (**b**) Quantitative RT-PCR analysis of *CXCL8*, *CXCL1* and *CXCL2* mRNAs in FTSECs expressing a control vector or GAB2. Cells were plated for 48 h before harvesting total RNA. Data are averages±s.e.m. from three independent experiments. *P<0.05; **P<0.01. (**c**) Quantitative RT–PCR analysis of *CXCL8*, *CXCL1* and *CXCL2* mRNAs in FUOV1 cells expressing Dox-inducible control shRNA or two independent shRNAs targeting GAB2. Cells were cultured in the absence or presence of 1 μg/ml Dox for 72 h with the last 24 h cultured in serum-free media. Data are averages±s.e.m. from three independent experiments. *P<0.05; **P<0.01. (**d**) Quantitative RT–PCR analysis of *CXCL8*, *CXCL1* and *CXCL2* mRNAs in FUOV1, NIH:OVCAR3 and IGROV1 ovarian cancer cells 48 h after transduction with a control shRNA targeting LacZ or two independent shRNAs targeting GAB2. Cells were cultured in serum-free media for the last 24 h before harvesting total RNA. Data are averages±s.e.m. from three independent experiments. *P<0.05; **P<0.01. (**e**) CXCL1 levels and (**f**) CXCL8 levels in the conditioned media measured by ELISA. FUOV1, NIH:OVCAR3 and IGROV1 cells were transduced with a control shRNA or two independent shRNAs targeting GAB2 for 48 h and then cultured in serum-free media for 24 h. Data are averages±s.d. from three independent experiments. *P<0.05; **P<0.01.

To investigate the mechanisms by which GAB2 overexpression promotes angiogenesis, we examined the repertoire of secreted factors affected by GAB2 overexpression using antibody arrays that analyzed 1000 factors including cytokines, chemokines, growth factors and other proteins. We previously showed that overexpression of GAB2 in immortalized fallopian tube secretory epithelial cells (FTSECs that were immortalized by human telomerase reverse transcriptase and SV40 early region) induced anchorage-independent growth.^4^ We therefore used FTSECs in this experiment. We cultured FTSECs overexpressing GAB2 or a control vector in serum-deprived media (0.1% fetal bovine serum) for 48 h before collecting the cell culture supernatant for antibody array analyses. As shown in Figure 2a, increased levels of six factors, including CXCL1, CXCL2, CXCL8, CCL2, SLPI and IGFBP2, and decreased levels of EDA-A2 were detected in conditioned media derived from GAB2-overexpressing cells compared with control vector-expressing cells.

CXCL1, CXCL2 and CXCL8 are of particular interest because the upregulation of CXCL1 and CXCL8 has been associated with poor survival in patients with ovarian cancer.^20, 21^ CXCL1 and CXCL2 are 90% identical in their amino-acid sequences.^22^ Quantitative RT–PCR analyses showed that the mRNA levels of *CXCL1*, *CXCL2* and *CXCL8* were also significantly increased in GAB2-overexpressing FTSECs compared with control vector-expressing cells (Figure 2b). To examine whether high GAB2 levels in ovarian cancer cells are required for expression of CXCL1, CXCL2 and CXCL8, we suppressed GAB2 with inducible shRNAs in FUOV1 cells and observed that induced suppression of GAB2 decreased the mRNA levels of *CXCL1*, *CXCL2* and *CXCL8* compared with un-induced cells (Figure 2c). To confirm these findings, we tested two additional previously published shRNAs targeting GAB2 (shGAB2 #6 and #7) or a control shRNA into FUOV1 cells and 2 additional ovarian cancer cell lines that also overexpressed GAB2 (NIH:OVCAR3 and IGROV1 cells) (Supplementary Figure 1). NIH:OVCAR3 cells harbor amplification of GAB2. Suppression of GAB2 in all three ovarian cancer cell lines decreased the mRNA levels of *CXCL1*, *CXCL2* and *CXCL8* (Figure 2d and Supplementary Figure 3). Furthermore, using enzyme-linked immunosorbent assay, we detected a significant reduction of the levels of CXCL1 and CXCL8 proteins in the conditioned media derived from FUOV1, NIH:OVCAR3 and IGROV1 cells after GAB2 suppression compared with respective control shRNA-expressing cells (Figure 2e). Therefore, these findings indicate that overexpression of GAB2 in ovarian cancer cells upregulates expression of CXCL1, CXCL2 and CXCL8 at both the transcriptional and protein levels.

We next examined whether there are correlations between expression levels of *GAB2* and these three chemokines in 573 primary HGSOCs characterized by the Cancer Genome Atlas project at cBioPortal (http://www.cbioportal.org). We assigned primary tumors into high and low expression levels by using *z*-score threshold±0.7. We observed that tumors expressing high levels of *GAB2* showed statistically significant or tendency toward significant co-occurrence with tumors expressing high levels of *CXCL1*, *CXCL2* and *CXCL8*, though significance was reached in *CXCL2* (*P*=0.033, Fisher's exact test) and *CXCL8* (*P*<0.001) but not CXCL1 (*P*=0.198) (Supplementary Figures 4a and b). We next examined the expression levels of GAB2 and these chemokines in 50 ovarian cancer cell lines characterized by the cell line encyclopedia project. We divided cell lines into high or low expression lines by mean expression value from all cell lines. We observed that cell lines expressing high levels of GAB2 showed significant or tendency toward significant co-occurrence with cell lines expressing high levels of CXCL1 (*P*=0.0099, Fisher's exact test) and CXCL2 (*P*=0.0697), but no significant correlation was observed between *GAB2* and *CXCL8* (Supplementary Figure 4c). Together, these findings suggest that overexpression of GAB2 in ovarian cancers may contribute to upregulation of *CXCL1*, *CXCL2* and *CXCL8* expression in a context specific manner.

### GAB2-induced chemokines promote endothelial cell tube formation

**Figure 3:**
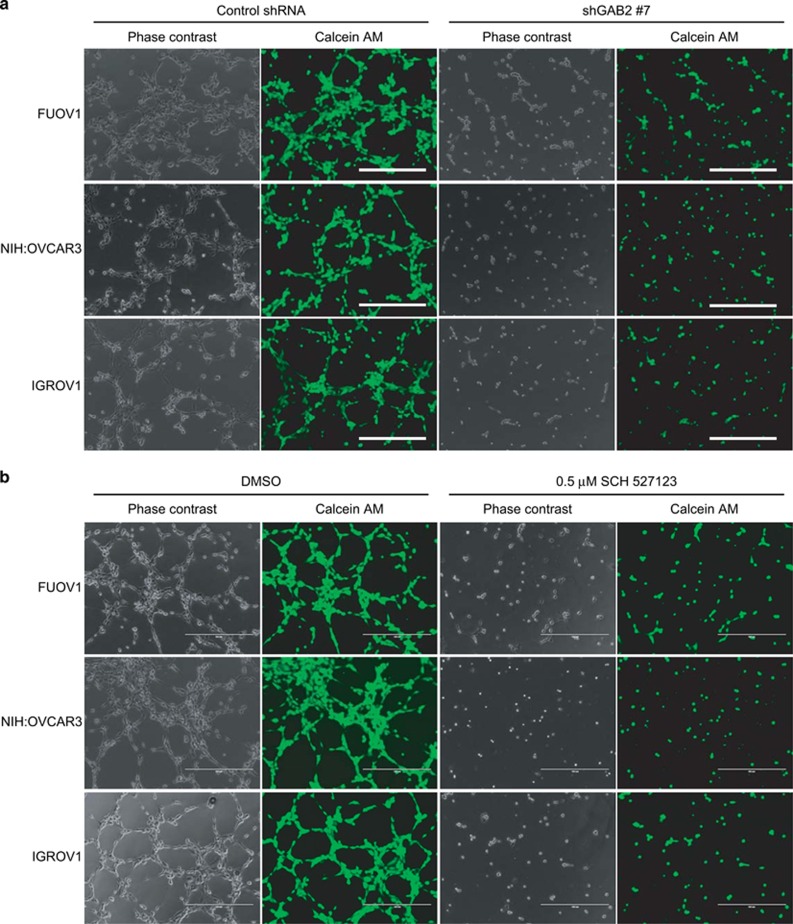
Effect of GAB2 suppression and CXCR2 blockade on tube formation of endothelial cells. (**a**) Representative phase contrast and green fluorescent Calcein AM images showing tube formation of HUVECs. Ovarian cancer cells were transduced with a control shRNA or a GAB2-targeting shRNA (shGAB2 #7) for 48 h and then cultured in serum-free media for 24 h. Conditioned media were added to HUVECs on matrigel and incubated for 8 h before imaging. Scale bar=400 μm. (**b**) Representative phase contrast and green fluorescent Calcein AM images showing the effect of CXCR2 antagonist on tube formation of HUVECs. Ovarian cancer cells were cultured in serum-free media for 24 h. Conditioned media were mixed with DMSO or 0.5 μm of SCH527123 before adding to HUVECs on matrigel and incubated for 8 h. Scale bar=400 μm. Representative images from two independent experiments were shown.

To further confirm whether GAB2-induced chemokines in ovarian cancer cells contribute to angiogenesis, we performed tube-formation assays using human umbilical vein endothelial cells (HUVECs) with matrigel. We introduced a control shRNA or a shRNA targeting GAB2 (shGAB2 #7) into FUOV1, NIH:OVCAR3 and IGROV1 cells, and determined whether conditioned media collected from these cells supported the tube formation of HUVECs. We observed that conditioned media from control shRNA-expressing cells induced tube formation of HUVECs (Figure 3a). By contrast, conditioned media from ovarian cancer cells after GAB2 suppression failed to support tube formation of HUVECs (Figure 3a). As the chemokines CXCL1, CXCL2 and CXCL8 signal through the receptor CXCR2, whereas CXCL8 can also bind to CXCR1, we also tested the effect of blocking CXCR2 by an antagonist SCH527123 on angiogenesis. We found that CXCR2 blockade abrogated the tube formation of HUVECs cultured in the presence of conditioned media from FUOV1, NIH:OVCAR3 and IGROV1 cells (Figure 3b). These results suggest that GAB2-regulated CXCL1, CXCL2 and CXCL8 in ovarian cancer cells display pro-angiogenic effect via CXCR2 on endothelial cells to induce tube-formation phenotype.

### GAB2-induced chemokines promote proliferation and survival of ovarian cancer cells

**Figure 4:**
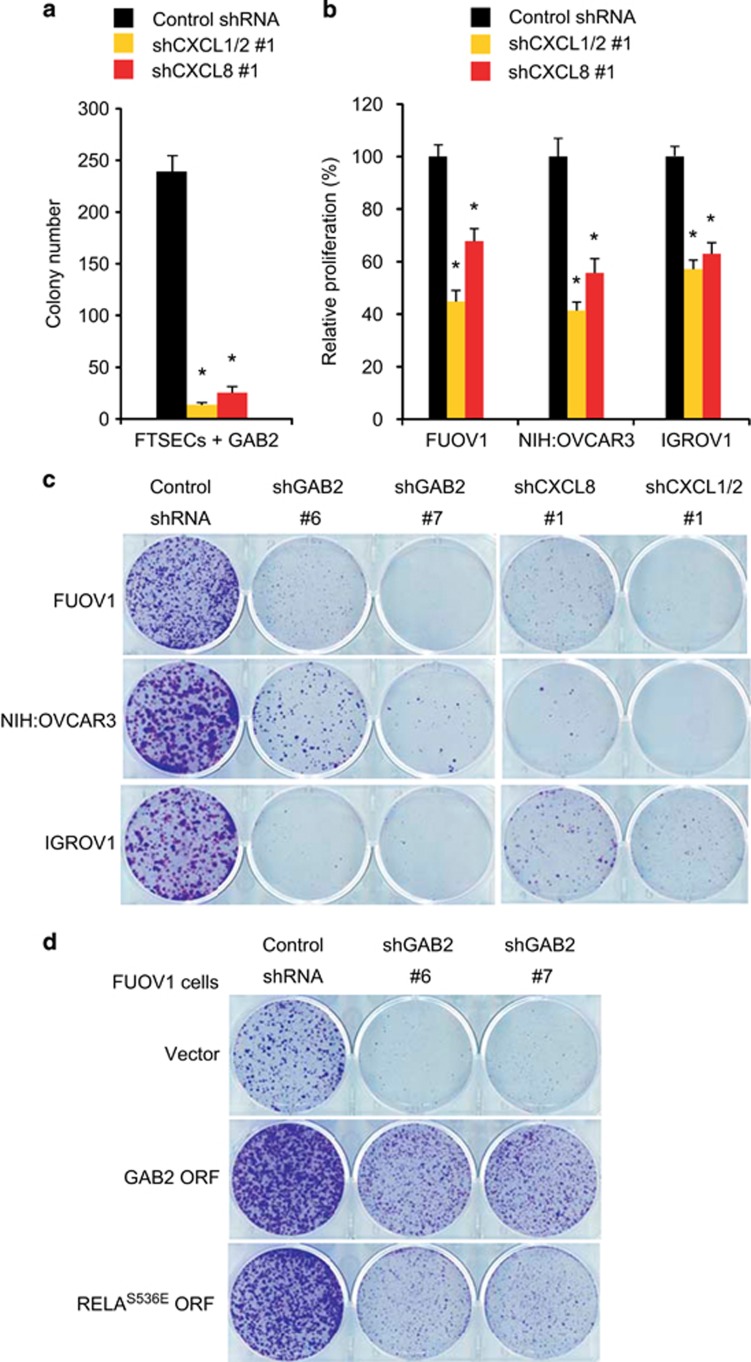
CXCL1, CXCL2 and CXCL8 are required for anchorage-independent growth of GAB2-transformed FTSECs and proliferation and survival of ovarian cancer cells. (**a**) Effect of suppressing CXCL1/2 or CXCL8 by shRNAs on anchorage-independent growth of GAB2-overexpressing FTSECs. Control shRNA targeting LacZ was used. Data are averages±s.e.m. from three independent experiments. *P<0.01. (**b**) Effect of suppressing CXCL1/2 or CXCL8 by shRNAs on the relative proliferation of FUOV1, NIH:OVCAR3 and IGROV1 ovarian cancer cells 5 days post transduction compared with cells infected with a control shRNA targeting LacZ. Data are averages±s.e.m. from three independent experiments. *P<0.01. (**c**) Effect of suppressing GAB2, CXCL8 or CXCL1/2 by shRNAs on clonogenic growth of FUOV1, NIH:OVCAR3 and IGROV1 cells. Representative images from two independent experiments were shown. (**d**) Effect of ectopic expression of GAB2 ORF or constitutively active RELAS536E ORF on clonogenic growth of FUOV1 cells upon GAB2 suppression. FUOV1 cells stably expressing GAB2 ORF, RELAS536E ORF or a control vector were infected with a control shRNA targeting LacZ or two independent shGAB2s (#6 or #7), which target the 3′-UTR region of endogenous *GAB2* mRNA, and assessed for clonogenic growth. Representative images from two independent experiments were shown.

Previous studies have suggested that CXCL1 and CXCL8 exhibit autocrine effects on ovarian cancer cell proliferation and survival.^21, 23, 24^ We therefore examined whether CXCL1, CXCL2 and CXCL8 are required for GAB2-induced transformation. We introduced a previously published shRNA^22^ that simultaneously targeted *CXCL1* and *CXCL2* (shCXCL1/2 #1), a shRNA targeting CXCL8 that induced >80% decrease in *CXCL8* mRNA levels (shCXCL8 #1) or a control shRNA targeting LacZ into GAB2-overexpressing FTSECs (Supplementary Figure 5). We found that suppression of CXCL1/2 or CXCL8 in GAB2-overexpressing FTSECs markedly abolished the anchorage-independent growth (Figure 4a), suggesting that the autocrine signaling generated by CXCL1/2 and CXCL8 contributes to GAB2-induced transformation. We next determined whether CXCL1, CXCL2 and CXCL8 were required for proliferation and survival of ovarian cancer cells with GAB2 overexpression. We found that suppression of CXCL1/2 or CXCL8 in FUOV1, NIH:OVCAR3 and IGROV1 cells significantly inhibited the cell proliferation and clonogenic growth that recapitulated the effect of GAB2 suppression (Figures 4b and c). To confirm the inhibitory effects of shGAB2s to be *GAB2* gene-specific, we performed a rescue experiment in which we transduce FUOV1 cells to stably overexpress GAB2 ORF or a control vector followed by infection with shGAB2s (which target the 3′-UTR of endogenous GAB2 mRNA). We observed that overexpression of GAB2 ORF in FUOV1 cells enhances clonogenic growth compared with control vector-expressing cells. Importantly, the overexpression of GAB2 ORF protected FUOV1 cells from the shGAB2-induced inhibition on clonogenic growth (Figure 4d). Taken together, in consonance with previous observations, our results suggest that CXCL1, CXCL2 and CXCL8 could act as autocrine growth factors that directly promote proliferation and survival of ovarian cancer cells that overexpressed GAB2.

### GAB2-induced chemokine expression is dependent on IKKβ-NF-κB signaling

**Figure 5:**
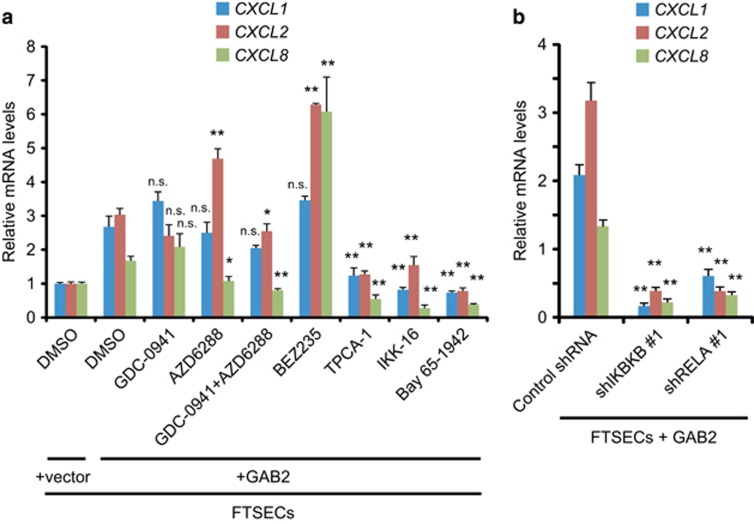
GAB2-induced chemokine expression is dependent on IKKβ-NF-κB activity. (**a**) Effect of small-molecule inhibitors against PI3K (GDC-0941), PI3K/mTOR (BEZ235), MEK (AZD6288) or IKKβ (TPCA-1, IKK16 and Bay 65–1942) on *CXCL1*, *CXCL2* and *CXCL8* mRNA levels in GAB2-overexpressing FTSECs. Cells were treated with 2 μm of each inhibitor or DMSO control for 6 h before collected for quantitative PCR analyses. Data are averages±s.e.m. of four independent experiments. Comparison between DMSO- or inhibitor-treated GAB2-overexpressing FTSECs were used for statistical analyses. n.s., not significant; *P<0.05; **P<0.01. (**b**) Effect of suppressing IKKβ (encoded by *IKBKB*) or NF-κB p65 (encoded by *RELA*) on *CXCL1*, *CXCL2* and *CXCL8* mRNA levels in GAB2-overexpressing FTSECs. Cells were infected with a control shRNA targeting LacZ or shRNAs targeting IKBKB or RELA and cultured for 48 h before collected for quantitative PCR analyses. Data are averages±s.e.m. of three independent experiments. *P<0.05; **P<0.01.

As PI3K and mitogen-activated protein kinases pathways represent two major effector pathways regulated by GAB2, we investigated if inhibition of PI3K or MEK could suppress GAB2-induced chemokine expression. GAB2-overexpressing FTSECs were treated with a pan-PI3K inhibitor (GDC-0941), a dual PI3K/mTOR inhibitor (BEZ235) and a MEK inhibitor (AZD6244) alone or in combination for 6 h and then collected for quantitative PCR analyses. To our surprise, inhibition of PI3K, PI3K/mTOR or MEK failed to suppress *CXCL1*, *CXCL2* and *CXCL8* mRNA levels compared with control treatment (Figure 5a). Previous studies suggest that IKKβ regulates the expression of CXCL1, CXCL2 and CXCL8 in ovarian cancer cells. We therefore tested the effect of several small-molecule inhibitors against IKKβ (TPCA-1, IKK16 or Bay 65–1942) on expression of these chemokines in GAB2-overexpessing FTSECs. Indeed, treatment with each of these IKKβ inhibitors could effectively reduce the mRNA levels of *CXCL1*, *CXCL2* and *CXCL8* after 6 h exposure (Figure 5a). To confirm these findings, we introduced shRNAs targeting IKKβ, NF-κB p65 subunit or a control shRNA targeting LacZ into GAB2-overexpressing FTSECs. In agreement with the results from small-molecule inhibitors, suppression of IKKβ or p65 also significantly reduced *CXCL1*, *CXCL2* and *CXCL8* mRNA levels compared with control shRNAs (Figure 5b and Supplementary Figure 6). Taken together, these results suggest that GAB2-induced chemokine expression could be effectively suppressed by inhibition of IKKβ-NF-κB pathway, but not inhibition of PI3K, mTOR or MEK.

We next determined whether NF-κB pathway has an important role downstream of GAB2 overexpression by assessing the effects of expressing constitutively active p65 mutant (S536E) in FUOV1 cells followed by GAB2 suppression. We found that overexpression of RELA^S536E^ in FUOV1 cells not only protected cells from the shGAB2-induced inhibition on clonogenic growth, but also enhanced clonogenic growth compared with control vector-expressing cells (Figure 4d). These results suggest that p65-dependent transcriptional activity is required for proliferation and survival of ovarian cancer cells that overexpresses GAB2.

### IKKβ inhibition increases sensitivity of ovarian cancer cells to PI3K/mTOR inhibition

**Figure 6:**
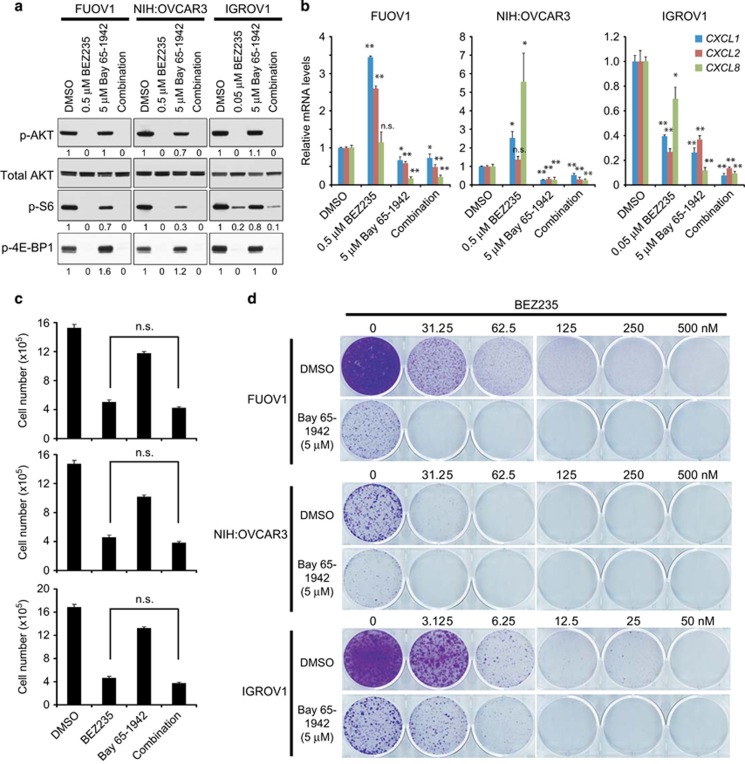
Effect of inhibition of PI3K/mTOR and IKKβ on proliferation and survival of ovarian cancer cells. (**a**) Effect of BEZ235 (a dual PI3K/mTOR inhibitor) and Bay 65–1942 (an IKKβ inhibitor) on activities of AKT, S6 and 4E-BP1. Immunoblots of p-AKT S473, p-S6 and p-4E-BP1 in FUOV1, NIH:OVCAR3 and IGROV1 ovarian cancer cells after exposure to indicated doses for 6 h. The values below the figures represent relative intensity of the bands normalized to total AKT levels and compared with DMSO-treated cells. (**b**) Effect of BEZ235 and Bay 65–1942 on *CXCL1*, *CXCL2* and *CXCL8* mRNAs in FUOV1, NIH:OVCAR3 and IGROV1 cells. Quantitative RT–PCR analysis was performed in cells treated with indicated inhibitors or DMSO control for 6 h. Data are averages±s.e.m. from three independent experiments. *P<0.05; **P<0.01. (**c**) Effect of BEZ235 and Bay 65–1942 on cell viability of FUOV1 (top panel), NIH:OVCAR3 (middle panel) and IGROV1 (bottom panel) cells after 72 h treatment. The same concentration of inhibitors as in panels **a** and **b** were used. The number of viable cells (unstained by trypan blue) was counted after 72 h treatment. Data are averages±s.e.m. of four independent experiments. n.s., not significant. (**d**) Effect of BEZ235 and Bay 65–1942 on clonogenic growth of ovarian cancer cells after exposure for 12–14 days. Representative images from two independent experiments are shown.

Increased CXCL8 secretion in breast cancer has been shown to mediate adaptive resistance to PI3K/mTOR-targeted therapy,^25^ whereas loss of phosphatase and tensin homolog protects breast cancer cells from CXCL8/CXCR1 inhibition,^26^ indicating that chemokine signaling may promote survival of cancer cells in response to PI3K-targeted therapy. We therefore tested the hypothesis that combined inhibition of both IKKβ and PI3K/mTOR pathways may inhibit proliferation and survival of ovarian cancer cells more effectively than individual inhibition. We first evaluated the effect of BEZ235 and Bay 65–1942 treatment individually or in combination on the PI3K/mTOR signaling and IKKβ target gene expression in FUOV1, NIH:OVCAR3 and IGROV1 ovarian cancer cells. We confirmed that BEZ235 treatment for 6 h effectively inhibited activities of PI3K/mTOR effectors as assessed by reduced p-AKT S473, p-S6 and p-4E-BP1 levels compared with control treatment (Figure 6a), whereas Bay 65–1942 treatment consistently reduced *CXCL1*, *CXCL2* and *CXCL8* mRNA levels in all three ovarian cancer cell lines tested (Figure 6b). By contrast, BEZ235 treatment reduced mRNA levels of these chemokines in IGROV1 cells but led to upregulation of CXCL1/2 or CXCL8 in FUOV1 and NIH:OVCAR3 cells, respectively (Figure 6b). We next evaluated the effect of PI3K/mTOR and IKKβ inhibition alone and in combination on proliferation and survival of ovarian cancer cells. We found that combining Bay 65–1942 with BEZ235 did not further reduce the number of viable ovarian cancer cells after 3-day treatment compared with BEZ235 alone. Although treatment with Bay 65–1942 alone only resulted in 20–30% inhibition on proliferation of three ovarian cancer cell lines after 3-day exposure (Figure 6c), we observed a pronounced inhibition on the clonogenic growth following exposure to Bay 65–1942 alone for 12–14 days (Figure 6d). Furthermore, combinatorial treatment with Bay 65–1942 and BEZ235 inhibited clonogenic growth of ovarian cancer cells more effectively than individual treatment (Figure 6d). These results suggest that co-targeting IKKβ and PI3K/mTOR is more effective in suppressing proliferation and survival of ovarian cancer cells than individual inhibition.

## Discussion

Patients with ovarian cancer are often diagnosed at advanced stage when tumors have spread into the peritoneal cavity. Although the standard therapy involves aggressive surgery followed by platinum/taxane-based chemotherapy, the majority of patients will experience relapse with chemo-resistant disease.^2^ Therefore, improved targeted therapies are urgently needed. This led the Cancer Genome Atlas projects to comprehensively characterize genetic abnormalities in primary HGSOCs to identify novel therapeutic targets.^3^ In parallel with such effort, we previously performed multiplexed *in vivo* transformation screens and identified GAB2 as a potent transforming gene.^4^ In addition to recurrent genomic amplification, GAB2 is overexpressed in one-third of primary HGSOCs.^4^ Here, we provided additional evidence showing that overexpression of GAB2 in ovarian cancer cells was required for peritoneal tumor growth by increasing tumor angiogenesis and cell proliferation. We found that overexpression of GAB2 in ovarian cancer cells upregulated expression of multiple chemokines, including CXCL1, CXCL2 and CXCL8 that exhibited mitogenic and pro-angiogenic activities. Taken together, these findings not only support our conclusion that GAB2 is a frequently altered oncogene in ovarian cancer but also assign GAB2 as an inducer of tumor angiogenesis important for disease development and progression.

Formation of new blood vessels is crucial for solid tumor growth and metastasis.^27^ Higher tumor microvessel density is associated with a shorter survival in patients with ovarian cancer. Tumor cells actively release pro-angiogenic factors such as vascular endothelial growth factor to promote endothelial cell proliferation, survival and migration for the formation of new blood vessels.^28^ In corroboration with previous findings showing that CXCL1 and CXCL8 are potent pro-angiogenic factors frequently upregulated in ovarian cancer,^20, 21^ we showed that CXCR2 blockade by antagonist SCH527123 completely inhibited the tube formation of HUVECs induced by ovarian cancer cells. Many CXCR2 antagonists are being investigated in clinical trials for chronic inflammatory diseases and have safe profiles with long-term usage.^29^ Another approach that coordinately targets these chemokines is to suppress IKKβ-NF-κB activity. We showed that inhibition of IKKβ by both genetic and pharmacological means effectively reduced the transcription of *CXCL1*, *CXCL2* and *CXCL8* in GAB2-overexpressing FTSECs and ovarian cancer cells. The paracrine signaling network induced by CXCL1/2 has recently been linked to cancer chemoresistance and metastasis in breast cancer.^22^ Further study is required to evaluate whether targeting CXCR2 or IKKβ alone will exhibit anti-angiogenic activity and also augment the efficacy of chemotherapy against ovarian cancer.

Elevated levels of CXCL1 and CXCL8 in serum, ascites and tumors have been associated with poor prognosis and shorter survival in patients with ovarian cancer.^20, 21, 30^ We showed that suppression of CXCL1/2 or CXCL8 significantly inhibited proliferation and clonogenic growth of GAB2-transformed FTSECs and ovarian cancer cells. Our findings are in agreement with previous studies showing that CXCL8 acts as a mitogenic factor and increases ovarian cancer cell proliferation, anchorage-independent growth and invasion, likely by activating AKT and ERK signaling.^24^ Silencing of CXCL8 with liposome-encapsulated siRNA inhibited ovarian tumor growth and angiogenesis.^20^ Recent study further revealed that autocrine CXCL8 signaling through the receptor CXCR1 in breast cancer is required for maintaining cancer stem cells,^26^ and CXCR1 blockade by antagonist reparixin selectively eliminated these cells and impaired tumor growth.^26^ Similarly, induction of CXCL1 has been demonstrated to be required for survival of RAS-transformed ovarian surface epithelial cells and ovarian cancer cells.^21^ Overexpression of CXCL1 or its primary receptor CXCR2 increases ovarian cancer cell proliferation in part by transactivation of EGFR signaling.^23, 31^ Blocking CXCL1 by a selective neutralizing antibody or suppression of CXCR2 by RNAi induces apoptosis in ovarian cancer cells.^21, 31^ The diverse roles of NF-κB signaling in ovarian cancer development and progression make it an attractive therapeutic target. Indeed, we showed that inhibition of IKKβ not only significantly reduced expression of *CXCL1*, *CXCL2* and *CXCL8*, but also suppressed clonogenic growth of ovarian cancer cells as a single agent. Importantly, we found that combinatorial inhibition of IKKβ and PI3K/mTOR could effectively abolish clonogenic growth of ovarian cancer cells compared with individual inhibition. As BEZ235 exhibits multifaceted anti-tumor activities in part by suppressing vascular endothelial growth factor-dependent angiogenesis,^32^ further study of this combination strategy in ovarian cancer is warranted to determine whether greater anti-angiogenic and anti-tumor effects could be achieved.

GAB2 is a scaffold adapter protein that lacks intrinsic enzymatic activities but mediates protein–protein interactions to transduce signals from receptors to diverse downstream effectors. Our prior study showed that GAB2 is amplified and/or overexpressed in approximately one-third of primary HGSOCs.^4^ We and others observed that ovarian cancer cell lines overexpressing GAB2 are sensitive to PI3K inhibition.^4, 14^ This study revealed an important role of GAB2 overexpression in promoting ovarian tumor growth and angiogenesis by upregulating IKKβ-dependent expression of CXCL1, CXCL2 and CXCL8. Therefore, amplification and overexpression of GAB2 may represent one of the early genetic events that promote ovarian tumor growth, and as a result, ovarian tumors overexpressing GAB2 exhibit dependence on high GAB2 levels for tumor growth. Recent studies have identified several mechanisms by which cancer cells adapt to PI3K-targeted therapy, such as reprogramming of mitochondrial trafficking^33^ and upregulation of prosurvival proteins.^17^ Although it remains challenging to directly target protein–protein interactions between GAB2 and effectors, our results suggest that co-targeting IKKβ and PI3K pathways downstream of GAB2 might be a promising therapeutic strategy for ovarian cancer that overexpresses GAB2.

## Materials and methods

### Plasmids

pLX304-blasticidin-GAB2, -GAB2Δp85 and pLX empty control vector have been described.^4^ pLenti6.2-blasticidin-luciferase has been described.^34^ RELA^S536E^ ORF was obtained from Addgene (Cambridge, MA, USA; #24156) and cloned into pLX304 vector. All pLKO.1-shRNA plasmids were designed by The RNAi Consortium with the following clone reference numbers or targeting sequences: control shRNA targeting LacZ (shLacZ) (TRCN0000231710), shGAB2 #6 (TRCN0000154991), #7 (TRCN0000155271), #9 (TRCN0000413156), #11 (TRCN0000415678), shCXCL1/2 #1 (TRCN0000057940), shCXCL8 #1 (TRCN0000232051), #2 (TRCN0000369255), #3 (TRCN0000058028), shIKBKB #1 (5′-TGGACAGTGTCCAATTCAAAT-3′), shRELA #1 (TRCN0000014684), #2 (TRCN0000014687), #3 (TRCN0000329800) and #4 (TRCN0000329877).

### Cell culture

FTSECs expressing human telomerase reverse transcriptase and the SV40 large T and small T antigens were provided by Dr Ronny Drapkin (Dana-Farber Cancer Institute, Boston), and cultured in DMEM/Ham's F12 50/50 mix (Corning, Corning, NY, USA) supplemented with 10% fetal bovine serum (Corning). Additional introduction of GAB2 or a control vector into FTSECs were described previously.^4^ FUOV1, NIH:OVCAR3 and IGROV1 cells were obtained and cultured as described.^35^ These cell lines have been authenticated by sequenom genotyping assays for a panel of 48 single-nucleotide polymorphism loci with reference to the established fingerprint (http://www.broadinstitute.org/ccle). No mycoplasma contamination was detected.

### Chemicals

Bay 65–1942 was purchased from ChemScene. GDC-0941, BEZ235, AZD6244, TPCA-1 and IKK16 were purchased from Selleck Chemicals (Houston, TX, USA).

### Antibody array detection

In total, 1 × 10^6^ of FTSECs expressing GAB2 or a control vector were plated into 10-cm culture dishes for 24 h. The media were replaced with serum-deprived media containing 0.1% fetal bovine serum, and the cells were cultured for 48 h. The culture supernatants were collected, centrifuged at 1000 × g, and dialyzed with 2 l of 1 × phosphate-buffered saline (pH 8) twice for overnight at 4^o^C. Samples were labeled with biotin and incubated with Human L1000 Antibody Arrays (#AAH-BLM-1000, RayBiotech, Norcross, GA, USA). Representative images from two independent experiments were shown.

### Cell proliferation assays

For assessing cell proliferation, 2000 of FUOV1, 4000 of NIH:OVCAR3 and 1500 of IGROV1 cells were seeded into each well of 96-well plates for 24 h. Six replicate infections were performed for control shLacZ or each gene-specific shRNA in the presence of 4 μg/ml polybrene for 24 h. Media were then replaced with fresh media with three replicate wells containing 2 μg/ml puromycin. After 5 days, the cell viability was measured by CellTiter-Glo luminescent cell viability assay (Promega, Madison, WI, USA). Data represent averages±s.e.m. of three independent experiments.

For assessing clonogenic growth, 10 000 of FUOV1, 8000 of NIH:OVCAR3 and 3000 of IGROV1 cells were seeded into each well of six-well plates for 24 h. Infections were performed for control shLacZ or each gene-specific shRNA in the presence of 4 μg/ml polybrene for 24 h. Media were replaced with fresh media containing 2 μg/ml puromycin. After 12–14 days, cells were fixed in 2.5% of buffered formalin for 15 min and stained with 0.1% (w/v) crystal violet, 20% (v/v) ethanol solution for 15 min. After several rinses in tap water and air-drying, plates were scanned by an Epson photo scanner. Representative images from two independent experiments were shown.

### Anchorage-independent growth assay

Growth in soft agar was determined by plating 5 × 10^4^ cells in triplicate in 0.4% Noble agar. Colonies >0.1 mm in diameter were counted 4 weeks after plating. Data represent averages±s.e.m. of three independent experiments.

### Endothelial cell tube-formation assay

Primary HUVECs were purchased from Life Technologies (Carlsbad, CA, USA) and cultured in medium 200PRF supplemented with large vessel endothelial supplement. Trypsinized HUVECs were resuspended in conditioned media derived from ovarian cancer cells and seeded in growth factor reduced matrigel (Corning) for 6 h before staining with Calcein AM (Life Technologies) for imaging.

### Tumorigenicity assay

In total, 5 × 10^6^ each of FUOV1+Luc cells expressing pLKO-TetOn-Control shRNA or pLKO-TetOn-shGAB2 #11 were resuspended in 200 μl of 1 × phosphate-buffered saline, and injected intraperitoneally into each of 6-week-old female athymic nude mice (Harlan Laboratories, Indianapolis, IN, USA). Noninvasive bioluminescent imaging was performed at 17, 52, 59 and 66 days post implantation. Doxycycline-containing diet (#TD.01306) was purchased from Harlan Laboratories. Five mice per group were followed for tumor growth and luminescence signals were compared via two-sided *t*-test. With five mice per group, the *t*-test has 90% power to detect a difference between groups of approximately 2.3 s.d.s. One mouse was excluded from the study because the luminescence signals detected before shRNA induction were too low. No randomization of animals was used for these groups. The investigator responsible for bioluminescent imaging was blinded to the group allocation. Animal experiments were in compliance with ethical regulations approved by Institutional Animal Care and Use Committee at Medical University of South Carolina.

### Immunoblotting

Cell lysates were prepared in radioimmunoprecipitation assay lysis buffer supplemented with Halt Protease and Phosphatase Inhibitor Cocktail (Pierce, Waltham, MA, USA). Protein concentration was measured by the BCA Protein Assay kit (Pierce). Equal amount of protein (30 μg) was separated by NuPAGE Novex Bis-Tris 4–12% gels (Life Technologies) and transferred onto a nitrocellulose membrane using iBlot Gel Transfer Device (Life Technologies). The membrane was incubated with primary antibodies for 2 h at room temperature. Antibodies against p-AKT, p-ERK1/2, p-IκBα, p-RELA, p-S6, total AKT, ERK1/2 and GAB2 were purchased from Cell Signaling Technology. After incubation with the appropriate horseradish peroxidase linked secondary antibodies (Bio-Rad, Hercules, CA, USA) for 2 h at room temperature, the membrane was incubated with Enhanced Chemiluminescence Plus substrate (Pierce) and signals were detected by Pierce CL-Xposure Film. Expression of β-actin was assessed as an internal loading control by use of a specific antibody (sc-8432-HRP; Santa Cruz Biotechnology, Dallas, TX, USA). The intensity of bands was quantified by using Fiji image processing software (http://fiji.sc/Fiji).

### Enzyme-linked immunosorbent assay

FUOV1, NIH:OVCAR3 and IGROV1 cells were transduced with a control shRNA or two independent shRNAs targeting GAB2 for 48 h and then cultured in serum-free media for 24 h. The levels of CXCL1 and CXCL8 proteins in the cell culture supernatants were measured by using enzyme-linked immunosorbent assay kits from RayBiotech.

### Real-time quantitative reverse-transcription PCR

Total RNA was extracted with TRIzol reagent (Life Technologies), and 2 μg was used to synthesize the first-strand complementary DNA using Maxima First Strand complementary DNA Synthesis Kit (Thermo Scientific, Waltham, MA, USA). Quantitative PCRs were performed with Maxima SYBR Green qPCR Master Mix (Thermo Scientific). The primer sequences used were: *GAPDH* (5′-CCTGTTCGACAGTCAGCCG-3′, 5′-CGACCAAATCCGTTGACTCC-3′), *CXCL1* (5′-GCGCCCAAACCGAAGTC-3′, 5′-TGCAGGATTGAGGCAAGCTT-3′), *CXCL2* (5′-CTGCGCCCAAACCGAAGTCATA-3′, 5′-CTGCGCCCAAACCGAAGTCATA-3′), *CXCL8* (5′-CCTGATTTCTGCAGCTCTGT-3′, 5′-AACTTCTCCACAACCCTCTG-3′) and *IKBKB* (5′-GGAAGTACCTGAACCAGTTTGAG-3′, 5′-GCAGGACGATGTTTTCTGGCT-3′). For each experiment, triplicate reactions for each primer set were performed separately on the same complementary DNA samples using Roche LightCycler 480 II PCR instrument. The mean cycle threshold was used for the comparative cycle threshold analysis (ABI User Bulletin #2). Data represent averages±s.e.m. of three independent experiments.

### Statistical analysis

Two-tailed, unpaired Student's *t*-test was used for comparisons using Prism GraphPad software. *P*<0.05 was considered statistically significant. Similar variances between groups were observed by F-test.
