# Supplementary figures and images for: Overexpression of GAB2 in ovarian cancer cells promotes tumor growth and angiogenesis by upregulating chemokine expression
Source: Oncogene. 2015 Dec 14;35(31):4036–47. doi: 10.1038/onc.2015.472 (PMC4977484; doi:10.1038/onc.2015.472)

Supplementary Figure 1

**a**

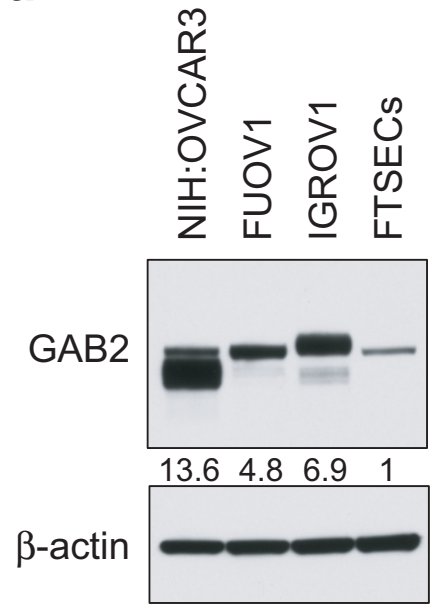

**b**

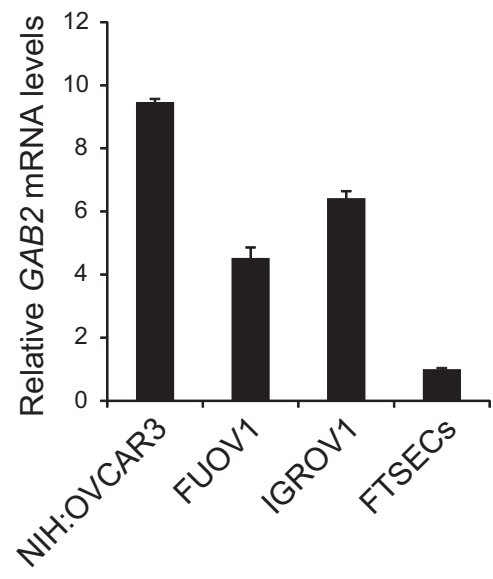

Supplement: Supplementary Figure 1 [file onc2015472x1.pdf]

## Supplementary Figure 2

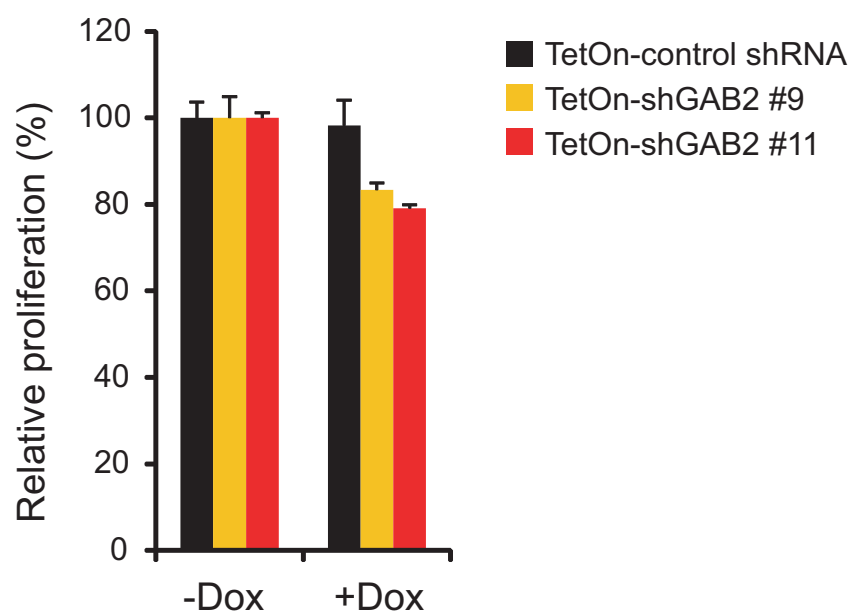

Supplement: Supplementary Figure 2 [file onc2015472x2.pdf]

# Supplementary Figure 3

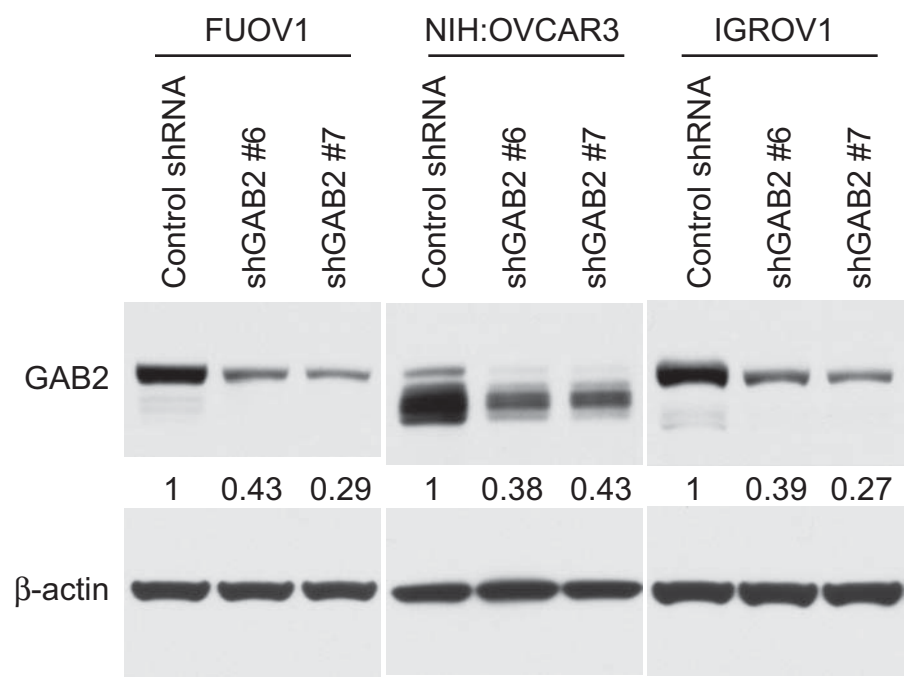

Supplement: Supplementary Figure 3 [file onc2015472x3.pdf]

Supplementary Figure 5

**a**

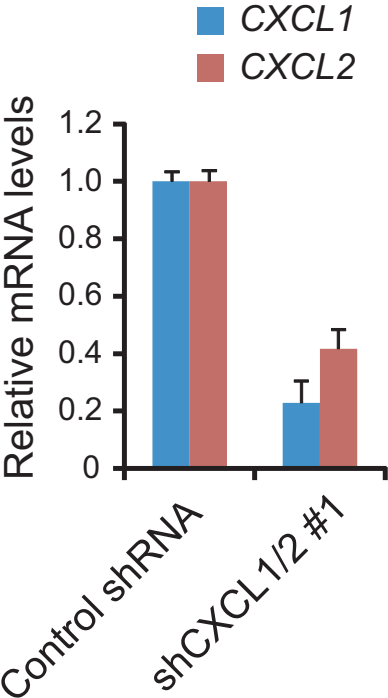

**b**

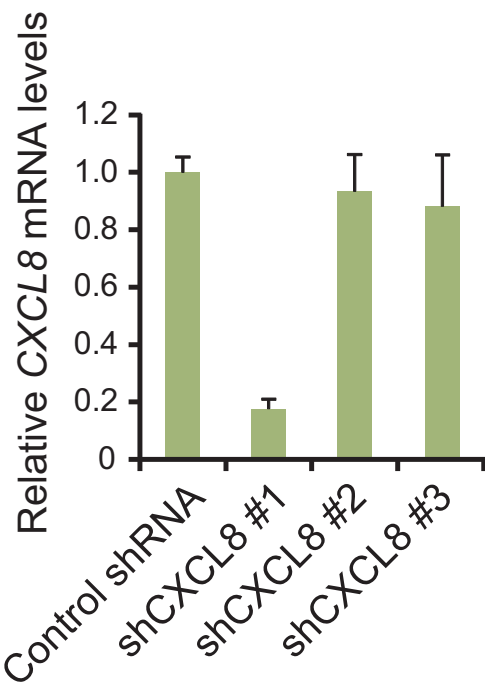

Supplement: Supplementary Figure 5 [file onc2015472x5.pdf]

Supplementary Figure 6

**a**

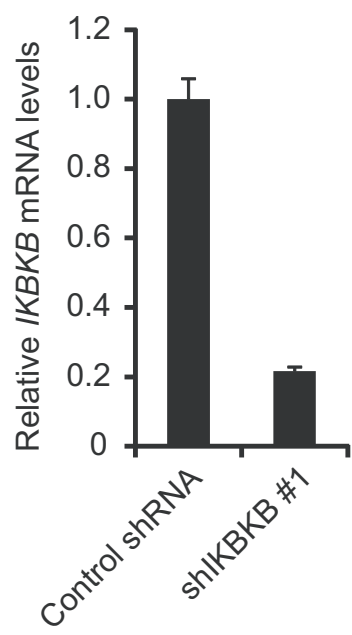

**b**

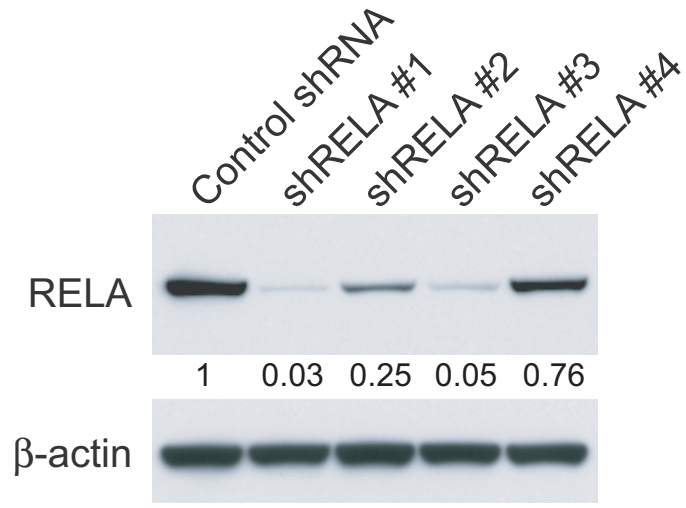

Supplement: Supplementary Figure 6 [file onc2015472x6.pdf]
