# Supplementary material for: Overexpression of GAB2 in ovarian cancer cells promotes tumor growth and angiogenesis by upregulating chemokine expression
Source: Oncogene. 2015 Dec 14;35(31):4036–47. doi: 10.1038/onc.2015.472 (PMC4977484; doi:10.1038/onc.2015.472)
Supplement: Supplementary Figure 4 [file onc2015472x4.pdf]

# Supplementary Figure 4

**a**

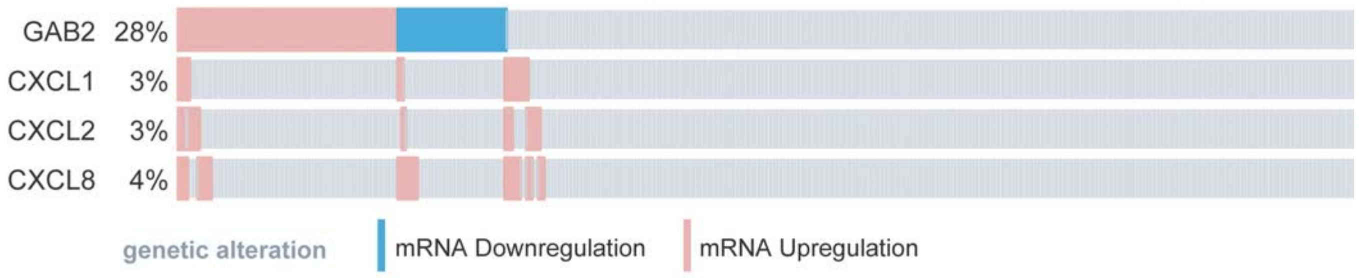


**b**

| Gene A | Gene B | p-Value | Log Odds Ratio | Association |
|---|---|---|---|---|
| GAB2 | CXCL1 | 0.198 | 0.539 | Tendency towards co-occurence |
| GAB2 | CXCL2 | 0.033 | 1.008 | Tendency towards co-occurence (significant) |
| GAB2 | CXCL8 | <0.001 | 1.521 | Tendency towards co-occurence (significant) |
| CXCL1 | CXCL2 | <0.001 | >3 | Tendency towards co-occurence (significant) |
| CXCL1 | CXCL8 | <0.001 | >3 | Tendency towards co-occurence (significant) |
| CXCL2 | CXCL8 | <0.001 | 2.659 | Tendency towards co-occurence (significant) |

**c**

| | CXCL1 | | CXCL2 | | CXCL8 | |
|---|---|---|---|---|---|---|
| | low levels | high levels | low levels | high levels | low levels | high levels |
| GAB2 - low levels | 19 | 8 | 5 | 22 | 11 | 16 |
| GAB2 - high levels | 7 | 16 | 10 | 13 | 10 | 13 |
| | *P*=0.0099 | | *P*=0.0697 | | Not significant | |
