# Supplementary material for: Overexpression of GAB2 in ovarian cancer cells promotes tumor growth and angiogenesis by upregulating chemokine expression
Source: Oncogene. 2015 Dec 14;35(31):4036–47. doi: 10.1038/onc.2015.472 (PMC4977484; doi:10.1038/onc.2015.472)
Supplement: Supplementary Figure Legends [file onc2015472x7.docx]

**Supplementary Figure legends**

**Supplementary Figure 1. Overexpression of GAB2 protein and mRNA levels in ovarian cancer cell lines.** (**a**) Immunoblots of GAB2 and β-actin control in ovarian cancer cell lines (NIH:OVCAR3, FUOV1, and IGROV1) and immortalized fallopian tube secretory epithelial cells (FTSECs). NIH:OVCAR3 cells harbor amplification of GAB2 gene, whereas FUOV1 and IGROV1 cells express high GAB2 levels but do not have amplification of GAB2. The values below the figures represent relative intensity of the bands normalized to β-actin compared to FTSECs. (**b**) Quantitative PCR analysis of GAB2 mRNA levels in indicated cell lines. Data are averages ± SD of triplicate measurements representative of 2 independent experiments.

**Supplementary Figure 2. Suppression of GAB2 by doxycycline-inducible shRNAs inhibited cell proliferation of FUOV1 cells.** FUOV1+Luc cells expressing Dox-inducible control shRNA or 2 previously published shRNAs targeting GAB2 at different regions were plated in the presence or absence of 1 μg/mL of doxycycline for 6 days. The relative proliferation of doxycycline-treated cells were measured by Promega Steady-Glo Luciferase assay, and were normalized to untreated cells. Data represent average ± SD of triplicate measurements.

**Supplementary Figure 3. Validation of shRNAs that efficiently suppressed expression of GAB2.** Immunoblots of GAB2 and β-actin control in FUOV1, NIH:OVCAR3, and IGROV1 ovarian cancer cells 4 days after transduction with 2 independent shRNAs targeting GAB2 or a control shRNA targeting LacZ. The values below the figures represent relative intensity of the bands normalized to β-actin and compared to control shRNA-expressing cells.

**Supplementary Figure 4. Analysis of correlations between expression of GAB2, CXCL1, CXCL2, and CXCL8 in primary HGSOCs and 50 ovarian cancer cell lines.** (**a**) Expression of GAB2, CXCL1, CXCL2, and CXCL8 in 573 primary HGSOCs characterized by the TCGA project. Data were obtained from cBioPortal in which z-score threshold of 0.7 ± was applied to define high (blue color) or low expression levels (pink color) in each tumor (grey color). (b-c) Association between expression levels of GAB2, CXCL1, CXCL2, and CXCL8 in (**b**) 573 primary HGSOCs characterized by the TCGA project and (**c**) 50 ovarian cancer cell lines characterized by the cancer cell line encyclopedia project. Fisher’s exact test was used to determine statistical significance.

**Supplementary Figure 5. Validation of shRNAs that efficiently suppressed expression of CXCL1/2 or CXCL8.** Quantitative PCR analyses of (**a**) *CXCL1* and *CXCL2* and (**b**) *CXCL8* mRNA levels in GAB2-overexpressing FTSECs 3 days after transduction with a control shRNA targeting LacZ or shRNAs targeting CXCL1/2 or CXCL8. Data are averages ± SEM of 3 independent experiments.

**Supplementary Figure 6. Validation of shRNAs that efficiently suppressed expression of IKBKB or RELA.** (**a**) Quantitative PCR analyses of *IKBKB* mRNA levels in GAB2-overexpressing FTSECs 3 days after transduction with a control shRNA targeting LacZ or a shRNA targeting IKBKB. Data are averages ± SD of 3 independent experiments. (b) Immunoblots of RELA and β-actin control in GAB2-overexpressing FTSECs 4 days after transduction with 4 distinct shRNAs targeting RELA. The values below the figures represent relative intensity of the bands normalized to β-actin.
